# Transcriptome analysis of an incompatible *Persea americana-Phytophthora cinnamomi* interaction reveals the involvement of SA- and JA-pathways in a successful defense response

**DOI:** 10.1371/journal.pone.0205705

**Published:** 2018-10-17

**Authors:** Noëlani van den Berg, Waheed Mahomed, Nicholas A. Olivier, Velushka Swart, Bridget G. Crampton

**Affiliations:** 1 Department of Biochemistry, Genetics and Microbiology, University of Pretoria, Pretoria, Gauteng, South Africa; 2 Forestry and Agricultural Biotechnology Institute (FABI), University of Pretoria, Pretoria, Gauteng, South Africa; 3 Department of Plant and Soil Sciences, University of Pretoria, Pretoria, Gauteng, South Africa; 4 African Centre for Gene Technologies Microarray Facility, University of Pretoria, Pretoria, Gauteng, South Africa; Universidade de Lisboa Instituto Superior de Agronomia, PORTUGAL

## Abstract

*Phytophthora cinnamomi* Rands (*Pc*) is a hemibiotrophic oomycete and the causal agent of Phytophthora root rot (PRR) of the commercially important fruit crop avocado (*Persea americana* Mill.). Plant defense against pathogens is modulated by phytohormone signaling pathways such as salicylic acid (SA), jasmonic acid (JA), ethylene (ET), auxin and abscisic acid. The role of specific signaling pathways induced and regulated during hemibiotroph-plant interactions has been widely debated. Some studies report SA mediated defense while others hypothesize that JA responses restrict the spread of pathogens. This study aimed to identify the role of SA- and JA- associated genes in the defense strategy of a resistant avocado rootstock, Dusa in response to *Pc* infection. Transcripts associated with SA-mediated defense pathways and lignin biosynthesis were upregulated at 6 hours post-inoculation (hpi). Results suggest that auxin, reactive oxygen species (ROS) and Ca^2+^ signaling was also important during this early time point, while JA signaling was absent. Both SA and JA defense responses were shown to play a role during defense at 18 hpi. Induction of genes associated with ROS detoxification and cell wall digestion (β-1-3-glucanase) was also observed. Most genes induced at 24 hpi were linked to JA responses. Other processes at play in avocado at 24 hpi include cell wall strengthening, the formation of phenolics and induction of arabinogalactan, a gene linked to *Pc* zoospore immobility. This study represents the first transcriptome wide analysis of a resistant avocado rootstock treated with SA and JA compared to *Pc* infection. The results provide evidence of a biphasic defense response against the hemibiotroph, which initially involves SA-mediated gene expression followed by the enrichment of JA-mediated defense from 18 to 24 hpi. Genes and molecular pathways linked to *Pc* resistance are highlighted and may serve as future targets for manipulation in the development of PRR resistant avocado rootstocks.

## Introduction

Avocado is susceptible to Phytophthora root rot (PRR) caused by the soil-borne oomycete, *Phytophthora cinnamomi* Rands (*Pc*). In countries where the pathogen is prevalent, growers are dependent on the use of tolerant/resistant rootstocks in combination with phosphite treatments and orchard management for sustainable production [1]. Currently no rootstocks with complete resistance are available; however, efforts to select promising material are on-going in South Africa, Israel and California. Selection is based on the phenotypic disease assessment of thousands of avocado plantlets inoculated with *Pc* followed by rigorous field trials. Dusa®, commercially released in 2004, is one of the successful rootstocks discovered in this manner in South Africa. In addition to being resistant to *Pc*, Dusa® is graft compatible with many scions and the combinations are high yielding.

Avocado, like most plants employs a myriad of strategies to withstand pathogen attack. Efforts to understand defense mechanisms against *Pc* have been undertaken in several plant species including avocado and eucalyptus. In *Arabidopsis*, response to the pathogen includes reactive oxygen species (ROS) induction, hypersensitive response (HR) activation, lignin synthesis and callose production [2]. Hallmarks of non-host resistance were activated in *Arabidopsis*, which included the activation of ethylene (ET) and jasmonic acid (JA) pathways rather than the salicylic acid (SA) pathway [2]. Phenylalanine ammonium lyase (PAL) activity and an increase of phenolic compounds have been identified in resistant interactions between *Corymbia calophylla* and *Pc* [3]. Similarly, studies on the *Pc* infection of *Zea mays* concluded that the resistance response is multi-faceted. The research comprised of histochemical analysis and transcriptional analysis and found that resistance was underpinned by ROS generation, callose deposition and antimicrobial compounds highlighting the diverse defense strategies employed by different plant species in response to the hemibiotroph, *Pc*.

Recently, the interaction between avocado and *Pc* was studied on a protein [4, 5], metabolite [6, 7] and transcriptome level [8, 9]. García-Pineda et al. (2010) investigated ROS formation and the role of nitric oxide (NO) against *Pc*. ROS were released 4 days post inoculation (dpi), while NO production increased 72 hpi in response to the pathogen. The authors hypothesized that the SA pathway is important in pathogen restriction and also demonstrated that externally applied SA was associated with decreased root colonization by the pathogen [6]. Host dependent defense responses against *Pc* are however evident from a similar study in *Arabidopsis* [10].

More recently, proteins expressed during *Pc* infection of the highly tolerant avocado cultivar G755 were profiled [4]. Proteins such as isoflavone reductase, glutathione *S*-transferase, abscisic acid stress ripening protein, cinnamoyl-CoA reductase and cinnnamoyl alcohol dehydrogenase were up-regulated from around 3 hpi. Genes representing enzymes of the phenylpropanoid isoflavonoid pathway were implicated in the avocado-*Pc* interaction. Although evidence suggested that the SA pathway is activated in the avocado-*Pc* interaction the exact role of phytohormones in this interaction have not yet been defined. In a preliminary transcriptome-wide study of another avocado-*Pc* interaction it was demonstrated that genes representing multiple pathways were temporally up-regulated over 48 hrs in a tolerant rootstock, illustrating that tolerance is complex, involving ROS, cell wall strengthening and various phytohormone pathways [8].

The role of signaling pathway(s) important in hemibiotrophic interactions is subject to debate. There are proponents of SA mediated defenses against hemibiotrophs [11–13] with opponents supporting the hypothesis that JA induced responses restrict the spread of the pathogen [14]. However, evidence exists that both SA and JA pathways are involved in plant defense strategies [15]. In the wheat-*Fusarium graminearum* interaction a biphasic response was observed *in planta* that comprised of SA and Ca^2+^ signaling during the first 6 hpi followed by JA signaling around 12 hpi [16]. Studies on the interactions of different *Phytophthora* species have shown conflicting results with regard to SA- and JA pathway activation. The *Arabidopsis*-*Phytophthora capsici* interaction showed that *Arabidopsis* SA signaling mutants displayed severely compromised resistance to *P*. *capsici* while resistance was attenuated in only two of the JA-insensitive mutants. The authors concluded that the SA pathway was more important in this interaction [17]. In the soybean- *Phytophthora sojae* interaction, SA was also found to be strongly induced [18]. In contrast to this, resistance to *Phytophthora parasitica* in *Arabidopsis* was dependent on JA/ET signaling in addition to SA signaling [19]. These studies have shown that defense strategies are trophic dependent; however recent studies utilizing 'omics' tools have identified numerous similarities between biotrophic and necrotrophic based plant responses. More evidence supports that SA and JA responses are trophic independent and that diverse signals converge on these two defense hormones [14].

Similarly, the avocado-*Pc* interaction is a complex system where previous studies have hinted that the host utilizes both the SA- and JA- pathways to combat the biotrophic and necrotrophic stages of this hemibiotrophic oomycete [8]. Here we provide evidence of SA- and JA-associated gene regulation in a resistant avocado in response to *Pc*. Microarray expression profiling of SA- and JA- treated roots were contrasted against *Pc* infected root material at 6, 18 and 24 hrs.

## Materials and methods

### Plant material and RNA isolation

One-year-old *Pc-*resistant clonal Dusa plants were provided by Westfalia Technological Services (WTS) (Tzaneen, South Africa). Plants were divided into five groups; one for *Pc* infection, two for SA and MeJA treatments, respectively and two untreated, uninfected control groups (the first for the SA treatment and *Pc* treatments and the second for the MeJA treatment). Each group comprised of three time points (6, 18 and 24 hrs) and each time point had three biological replicates with two or three plants per replicate, therefore each time point had a minimum of six plants. Prior to the experiment different SA and MeJA concentrations were assessed for phytotoxicity and the ability to induce pathway specific genes *phenylalanine ammonia lyase* (*PAL*), *pathogenesis-related group-5* (*PR-5*), *lipoxygenase 1* (*LOX1*), *jasmonate ZIM-domain* (*JAZ3*) and *pathogenesis-related group-4* (*PR-4*). Based on these results (not shown) plants treated with SA received 70 ml of a 5 mM NaSA solution (Sigma-Aldrich, Missouri, USA) and the MeJA treated plants received 70 ml of a 5 mM MeJA solution (Sigma-Aldrich). Inoculation was carried out by applying 20 ml of a *Pc* zoospore suspension (3x10^5^ spores/ml) and 70 ml *Pc* mycelial suspension to the soil at the base of each plant [20]. Uninfected and untreated control plants were included at all time points and were treated with distilled water. Roots were harvested at the respective time points, snap frozen in liquid nitrogen and stored at -80°C. Root material was flash frozen in liquid nitrogen and homogenized using the IKA Tube Mill control (IKA, Staufen, Germany). The *Pc* diagnostic screening was conducted by nested LPV3 amplification of genomic DNA (gDNA) extracted from inoculated root samples to confirm pathogen infection [21]. Results were visualized on a 2% TAE agarose gel under non-denaturing conditions. RNA was extracted using the CTAB method [8, 9] and purified with the RNeasy MinElute Cleanup Kit (Qiagen) with the inclusion of an on-column RNase-free DNase I treatment (Thermo Fischer Scientific). Genomic DNA contamination was assessed using intron-spanning flavone-3-hydroxylase (F3H) primers [8, 9] and quality analysis was done on a Bio-Rad Experion automated electrophoresis system (Bio-Rad, California, USA). A minimum RQI (RNA quality indicator) value of 7 was applied for all samples.

### Microarray analysis

An Agilent microarray containing a total of 9160 avocado transcripts and 465 *Pc* transcripts was employed to study gene expression in the resistant Dusa rootstock as previously described [22]. Microarray analysis was performed on three biological replicates of SA-, MeJA- and *Pc-*treated avocado root material at 6 and 18 hrs post treatment (hpt), with only MeJA and *Pc-*treated material also analyzed at 24 hpt. Three biological replicates of uninfected and untreated control material were included in the experiment. A common reference design with no dye swop was employed and the reference pool was created by combining 1.95 μg of RNA from each time point of all treatments and control samples. cDNA synthesis, Cy5 and Cy3 labelling and hybridization were carried out as previously described [22]. Microarray hybridization was based on the competitive hybridization design to measure relative gene expression and conducted according to the two-colour microarray-based gene expression analysis protocol (Agilent Technologies, California, USA).

### Microarray scanning, data capture and statistical analysis

Microarray slides were scanned with the Axon GenePix 4000B Scanner (Molecular Devices, CA, USA), and spot intensity values measured with the Axon GenePix 6.0 software. Features with unsuitable saturation (.20%) and signal-to-noise ratio (<3) values were removed from the analysis. Statistical analysis was performed using the LIMMA (Linear models for microarray data) package from the Bioconductor project in R version 3.1.0 (R Foundation for Statistical Computing, http://www.R-project.org). Background correction was performed using the normexp function with an offset of 50 [23] followed by separate inter-slide normalizations using robust-spline normalization [22]. Between-array normalization was performed using g-quantile normalization for a common reference design and standard pair-wise Pearson correlations (*r*) determined concordance between biological replicates. Differential expression of transcripts was evaluated by fitting a linear model through all data points (lmFit). A contrast matrix was used to evaluate the linear model for comparisons of interest, followed by an empirical Bayes (eBayes) correction to moderate the standard errors of the estimated log-fold changes. Finally, *P*-values were adjusted for false positives using the false discovery rate (FDR) function. All transcripts with an adjusted *P*-value ≤0.05 for the specific comparisons were considered as statistically significant. Analysis of differentially expressed transcripts in microarray data has been historically typified by selecting genes that have a *P*-value ≤0.05 and exhibit a two-fold change in expression. This method is not always the best approach and its applicability to certain experiments is questionable [24]. Taking into consideration that we were interested in patterns of expression we relaxed the selection criteria for differential expression to include genes with fold changes of ≥1.0 and ≤-1.0 combined with an adjusted *P*-value ≤0.05. Occasionally genes that did not meet the criteria were considered based on prior evidence of differential expression during defense and individually assessed. Fold change significance was used as the selection criteria for the microarray data and gene lists were filtered according to fold change prior to further analysis. Venn diagrams were drawn using Venn-diagram (http://bioinformatics.psb.ugent.be/webtools/Venn/). Hierarchical clustering, by average linkage, was performed using Multi Experiment Viewer (MeV) version 4.8.1. The dataset from this study are available from the NCBI’s Gene Expression Omnibus through GEO Series accession number GSE119635 according to MIAME guidelines.

### Functional annotation, clustering and pathway analysis

The complete list of array probes with associated gene identities and annotations was provided previously [22]. Briefly, the annotation was performed using the desktop cDNA Annotation System (dCAS) software [25]. This allowed non-redundant (NR), Gene Ontology (GO), EuKaryotic Orthologous Groups (KOG) databases to be queried and assigned to the array probes. Blast2GO software (B2G; http://www.blast2go.com) was also used to assign GO terms for biological processes, molecular functions and cellular components to the array probes. Hierarchical clustering (HCL) was performed using Multi Experiment Viewer (MeV) software [26]. HCL was performed for the statistically unadjusted datasets, then for *P-*value filtered datasets and lastly for the fold change filtered datasets.

### RT-qPCR

Validation of microarray data was performed using reverse transcriptase quantitative polymerase chain reaction (RT-qPCR) amplification. Single strand cDNA synthesis was performed using the ImProm-II^TM^ single strand cDNA synthesis kit (Promega Corporation, Madison, USA). RT-qPCR expression profiles of *LOX*, *PR-4*, *PR-1* and *JAZ3* genes were compared against their microarray-generated expression profiles. *Actin*, *18S* and *α1-Tubulin* were used as reference genes for normalization of RT-qPCR expression data [22]. Primers were designed using PerlPrimer v1.1.21 (http://perlprimer.sourceforge.net) and synthesized by Inqaba Biotec (Pretoria, South Africa) (**S1 Table**). Primer specificity was tested using conventional PCR and by assessing the melting curves. RT-qPCR was conducted in accordance with the Minimum Information for Publication of RT-qPCR Experiments (MIQE) guidelines [27]. Amplification was performed in a total reaction volume of 10 μl using SensiMix SYBR No-ROX kit (Bioline USA, Inc., Taunton, USA) in triplicate. Normalized relative quantities (fold change) for genes were calculated [28] and the relative fold changes of the RT-qPCR data was compared against the log_2_-transformed fold change data of the microarray data. Statistical significance of the RT-qPCR data was determined by one-way ANOVA followed by a Student’s t-test (JMP version 10.0.0; http://www.jmp.com/,SAS Institute, Inc.) at *P* < 0.05.

## Results and discussion

**Fig 1:**
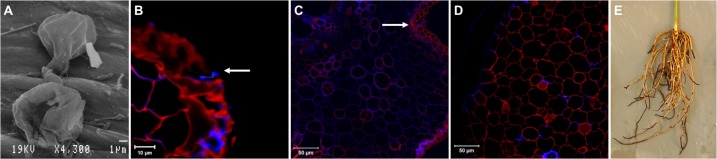
*Phytophthora cinnamomi* (*Pc*) infection of resistant avocado roots. **A.** Scanning electron micrograph of two *Pc* cysts germinating, with hyphae entering the root at the same site via direct penetration 1 hpi. **B.** Confocal image of *Pc* hyphae (white arrow) penetrating through the root epidermis at 3 hpi. **C.** Confocal image of lignified root cortex cells with hyphae (white arrow) at 12 hpi. **D.** Confocal image of root cortex cells demonstrating extensive lignification at 24 hpi. **E.** Brown necrotic lesions are visible on avocado roots six weeks after *Pc* inoculation.

*Phytophthora cinnamomi* zoospores typically encyst 1 hpi (**Fig 1A**) and hyphae can be observed penetrating the root epidermis at 3 hpi (**Fig 1B**). In response to *Pc* infection avocado cortex cells undergo lignification (**Fig 1C and 1D).** Resistant Dusa trees inoculated with *Pc* developed only mild root rot symptoms when assessed six weeks after inoculation (**Fig 1E**). Typical brown, necrotic lesions were visible, but as expected the majority of the roots were healthy. Pathogen identity and successful inoculation were confirmed by the amplification of a nested-PCR product of 77 bp (*Lpv3* gene) and by assessing morphological characteristics of the re-isolated *Pc* culture.

### The role of SA and JA signaling in response to *Pc* infection

The avocado stress microarray [9, 22] was employed to establish the role of SA- and JA- signaling pathways in the resistant avocado rootstock response to *Pc*. Gene expression profiles of *Pc*-infected root material was compared to profiles from SA- and MeJA- treated material at 6, 18 and 24 hrs. A total of 1093 transcripts were identified as being differentially expressed by applying a fold-change criterion of ≥1.0 or ≤-1.0 to the microarray data (**Table 1**) [29, 30]. The expression data were validated with RT-qPCR (**S1 Fig**).

**Table 1:** Number of differentially expressed avocado transcripts identified according to fold change on the microarray in response to salicylic acid (SA), methyl jasmonate (MeJA) and *Phytophthora cinnamomi* (*Pc)*.

Time points (hours)	Treatments		
	SA	MeJA	*Pc*
6 hrs up regulated	99	26	35
6 hrs down regulated	114	57	58
**Total 6 Hrs Gene Expression**	**213**	**83**	**93**
18 hrs up regulated	38	45	50
18 hrs down regulated	47	104	42
**Total 18 hrs gene expression**	**85**	**149**	**92**
24 hrs up regulated	N/A	65	119
**24 hrs down regulated**	N/A	26	168
Total 24 hrs gene expression	**N/A**	**91**	**287**
Total up regulated	137	136	204
Total down regulated	161	187	268
Total differential gene expression	298	323	472
**Total differential gene expression across all treatments**			**1093**

Duplicate transcripts were removed, and expression data were filtered using a >1.0 fold-change <-1.0. The three datasets included treatments with SA, MeJA and infection with *Pc*.

**Fig 2:**
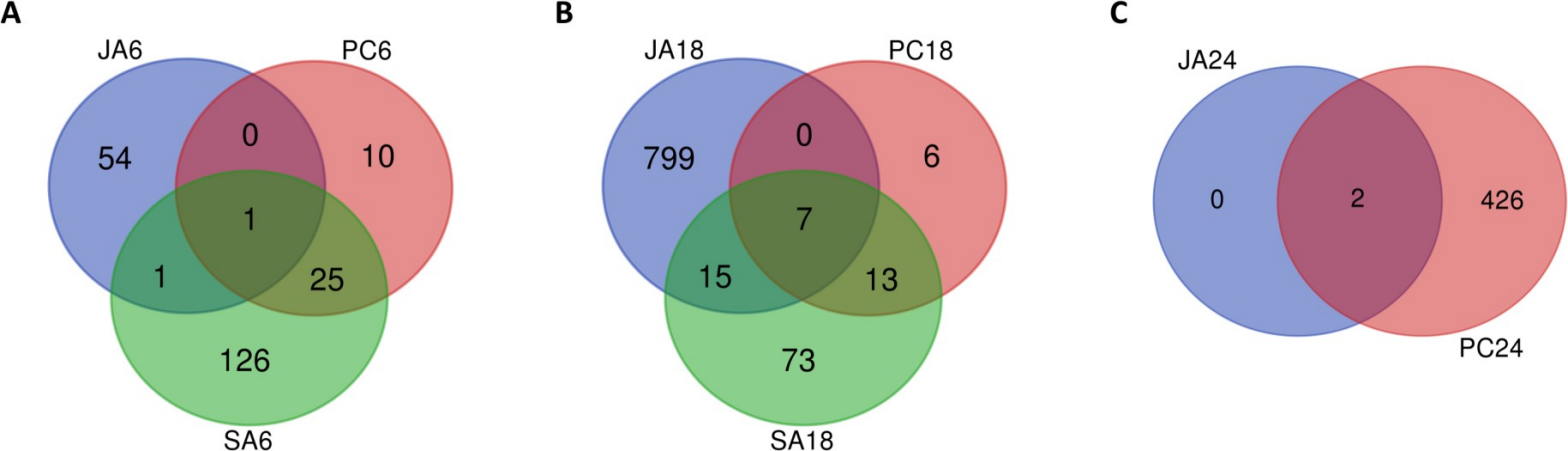
SA and JA-linked transcripts induced by *P*. *cinnamomi* (*Pc*) infection of avocado. Transcripts differentially expressed by either SA of MeJA treatments were identified in *P*. *cinnamomi* infected datasets. The values for the transcripts with multiple probes on the microarray was first averaged, and transcripts with poor Pearson correlation was removed. **A.** The total number of differentially expressed transcripts at 6 hours post treatment/infection. **B.** The total number of differentially expressed transcripts at 18 hours post treatment/infection. **C.** The total number of differentially expressed transcripts at 24 hours post treatment/infection. Note that no 24 hrs time point post SA treatment was analyzed.

Transcripts regulated by SA- and JA- signaling pathways during infection were identified by filtering all datasets according to the fold-change significant transcripts of infected datasets at 6, 18 and 24 hrs and identifying transcripts co-regulated in response to *Pc* and either SA or MeJA treatment. This resulted in 472 transcripts being identified as differentially expressed in response to *Pc*, with 204 transcripts being significantly up-regulated and 268 transcripts being significantly down-regulated in infected avocado roots. The number of differentially expressed transcripts common to *Pc* inoculated and phytohormone treated datasets at 6, 18 and 24 hours are shown in **Fig 2 (S2 Table)**. Transcripts regulated by SA are more prevalent at 6 hpt and genes regulated by JA are more prevalent at 24 hpt, which implies that SA- and JA- signaling pathways are inversely correlated and that infection by *Pc* causes the early induction of the SA pathway followed by the induction of the JA pathway. These findings were supported by hierarchical clustering (HCL) analysis.

**Fig 3:**
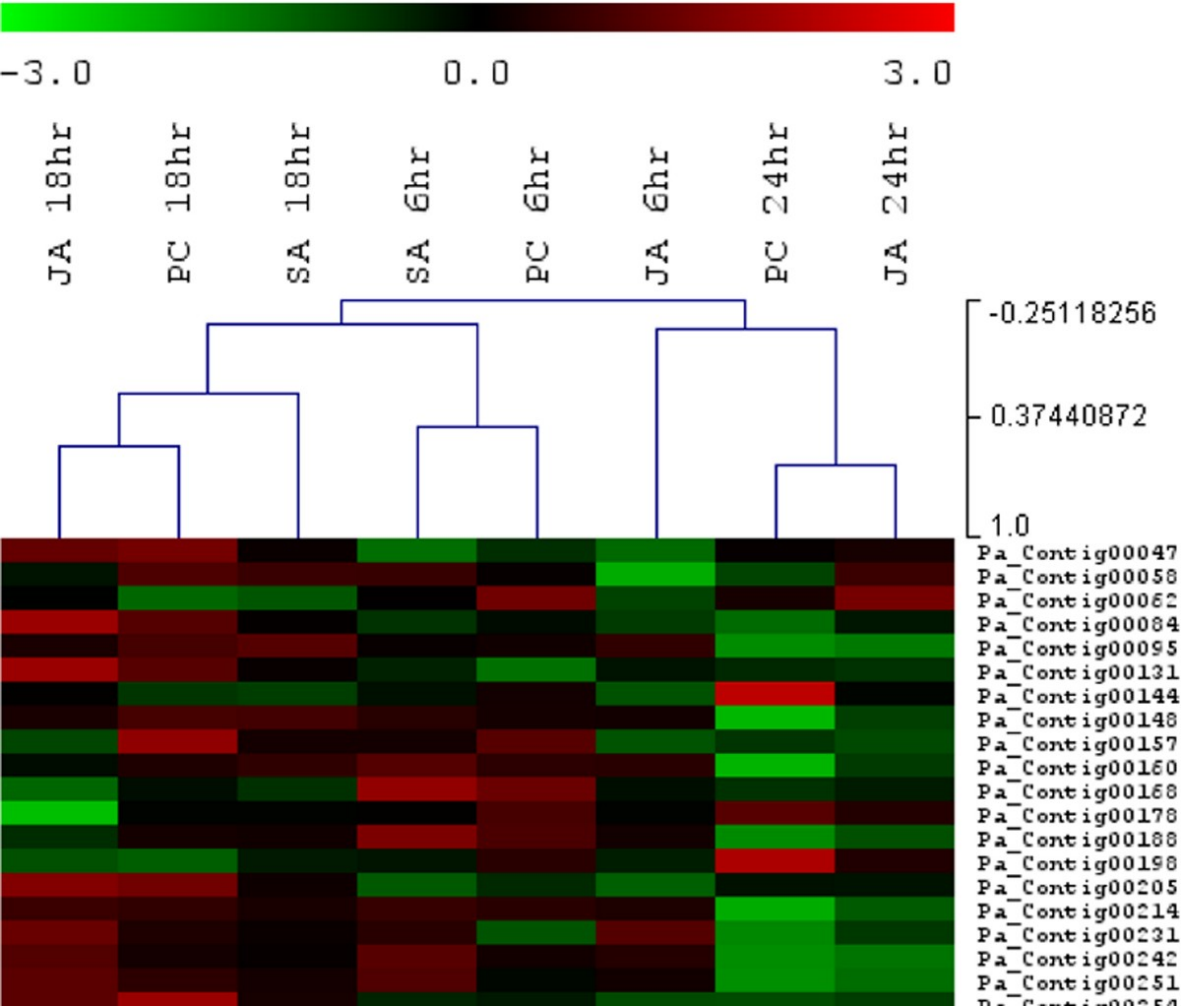
HCL performed on fold-change filtered avocado transcripts in the SA (salicylic acid), PC (*Phytophthora cinnamomi*) and JA (MeJA) datasets. The colour-scale indicates Log_2_ fold-change and the branches of the trees are ordered according to the Pearson correlation coefficient (*r*), with nodes closer to 1 indicative of a positive correlation.

HCL was performed on all significantly expressed transcripts (Log_2_ fold change, (≥1 and ≤-1)) in all *Pc*, SA and JA comparisons. A positive correlation was evident between the 6-hour post-*Pc* and 6-hour post-SA datasets. These two datasets were negatively correlated to all other 18-hour and 24-hour datasets (**Fig 3**). The 6-hour post-MeJA dataset displayed a negative correlation to all datasets. The 18-hour post-inoculation and post-MeJA datasets showed a positive correlation and grouped under one node while the 18-hour post-SA dataset grouped to these 18-hour datasets by an additional node. The 24-hour post-inoculation and post-MeJA datasets once again displayed a positive correlation to one another and were negatively correlated to all other datasets. Avocado transcripts known to be linked to SA signaling were regulated at 6 hrs in response to *Pc* inoculation. At 18 hrs transcripts from both SA and JA signaling pathways were present and at 24 hpi regulation of JA signaling was prevalent.

We propose that the initial induction of SA signaling and SA-mediated transcripts are in response to the biotrophic life stage of *Pc* [31]. Similar results are reported for the response in wheat to the hemibiotroph *F*. *graminearum* [16]. While the involvement of SA- and JA- signaling pathways in defense against hemibiotrophs has been debated [11–15], our findings suggest that the timeous regulation of both pathways are important in the avocado-*Pc* interaction.

### Processes in avocado roots affected by *Pc* infection

**Fig 4:**
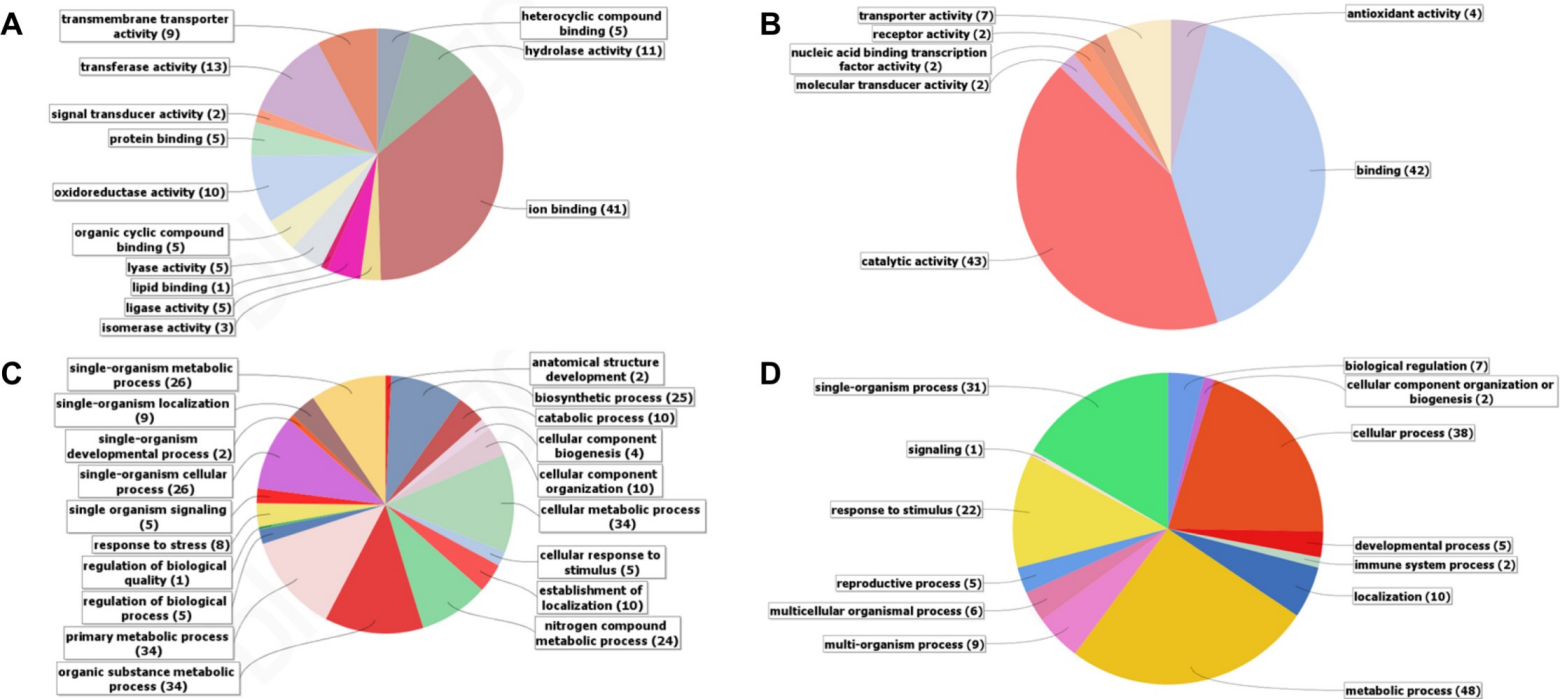
GO classification differentially expressed avocado transcripts in response to *Phytophthora cinnamomi* infection. **A**) Induced molecular function; **B**) repressed molecular function; **C**) induced biological process and **D**) repressed biological process. Second and third level GO terms are indicated with the corresponding percentage of transcripts for molecular function and biological processes respectively.

Several biological functions and molecular processes were affected by *Pc* as revealed by B2G annotation. The majority of induced avocado transcripts belonged to molecular processes of ion binding (41%), hydrolase activity (11%), transferase activity (13%) and oxidoreductase activity (10%) (**Fig 4A**). Biological processes induced by *Pc* were; responses to stress and stimulus (13%), single-organism metabolic processes (26%), single organism cellular processes (26%) and organic substance metabolic processes (34%) (**Fig 4B**). Pathways influenced by *Pc* inoculation included various metabolism pathways such as starch, glutamate, sugar and glycolysis. In addition to metabolic pathways the pentose phosphate, phenylpropanoid biosynthesis and oxidative phosphorylation pathways (represented by KEGG annotated enzymes) were also induced.

The majority of transcripts down-regulated in response to the pathogen were linked to processes of catalytic activity (43%) and binding (42%) (**Fig 4C**). The GO term “binding” included terms such as co-factor-, carbohydrate-, protein- and ion-binding. We observed a large component of biological process such as responses to stimulus (22%), metabolic processes (48%), single-organism processes (31%) and multi-organism processes (9%) being suppressed (**Fig 4D**). The GO term multi-organism processes are important in the infection context because it includes processes such as responses to other organisms, pathogenesis and cell adhesion, all of which are processes that a pathogen needs to manipulate the host to successfully infect.

GO analysis revealed that several cellular, biological and molecular processes were affected by pathogen infection. Ion binding was greatly induced at 6 hpi in response to *Pc*. Pathogen recognition is the first step in plant defense, followed by ion fluxes and signaling cascades, culminating in the activation of defense genes [32]. The Ca^2+^ influx of ions is induced by MAMPs, effectors, and hormones resulting in signal transduction and downstream activation of various signaling pathways [33]. At 18 hpi, GO biological processes were enriched for SA-induced chitin catabolism and ABA signaling as well as JA-induced cell wall macromolecule catabolism and biotic defense responses. SA-induced molecular functions such as peroxidase- and hydrolase-activities also featured in response to *Pc*. The increase in the GO term "oxidoreductase activity" is perceived as the activity of ROS during defense. The recognition of pathogen elicitors leads to ROS generation, phytoalexin production and defense gene induction [34]. Aside from its direct antimicrobial effects, ROS is widely accepted as a signal molecule in plants [35–37]. The induction of JA-induced defense responses such as chitin catabolism, auxin-related biological processes, Ca^2+^-dependent interactions and cell wall strengthening molecular functions continued at 24 hpi. The presence of both ABA- and auxin-induced processes at 18 hpi and 24 hpi along with SA and JA signaling are interesting but not unexpected [38, 39]. However, the significance of auxin and ABA in the avocado- *Pc* interaction remains unclear.

### Defense responses activated by *Pc* in avocado roots at 6, 18 and 24 hours post-inoculation

The top 25 differentially expressed transcripts at 6 hrs (**Table 2**), 18 hrs (**Table 3**) and 24 hrs (**Table 4**) post-*Pc* inoculation were selected and transcript regulation compared between the *Pc* dataset and the SA- and JA-treated datasets to identify common transcripts between the different treatments. SA signaling was strongly linked with *Pc* inoculation at 6 hrs, with 56% of the top 25 avocado transcripts up-regulated in response to *Pc* also induced by SA treatment. This declined to 40% at 18 hrs. Transcripts representing JA signaling accounted for 12% of induced transcripts in roots at 6 hpi and 36% at 18 hpi but increased to 72% at 24 hpi.

**Table 2:** Top 25 avocado transcripts differentially regulated by *Phytophthora cinnamomi* (*Pc*) at 6 hours post infection.

Sequence ID	Putative identification	Organism	6 hrs			18 hrs			24 hrs	
			SA	*Pc*	MeJA	SA	*Pc*	MeJA	*Pc*	MeJA
HA66E9C01ATMOZ	uncharacterized protein	*Arabidopsis thaliana*	2.47	2.64	1.26	-0.25	-0.03	0.60	2.24	3.35
GI32N0T02F7Q2J	predicted protein	*Hordeum vulgare* subsp. *vulgare*	1.24	2.04	0.77	1.24	0.76	-1.13	-0.71	-1.03
04939	metallothionein-like protein-like	*Vitis vinifera*	0.30	1.91	0.34	1.53	-0.02	-2.06	2.44	2.52
01026	polyphenoloxidase	*Camellia nitidissima*	0.66	1.88	0.61	0.08	-0.30	0.96	1.15	1.93
05008	putative polyphenol oxidase	*Dasiphora fruticosa*	0.57	1.66	0.42	-0.12	-0.32	0.92	0.72	1.66
02842	Annexin D5	*Triticum urartu*	1.01	1.66	0.79	1.49	0.60	-0.80	-0.52	-1.00
01719	Basic blue protein	*Medicago truncatula*	1.12	1.53	-0.50	-0.38	0.97	0.28	1.13	0.96
01120	PREDICTED: ribonuclease 1-like	*Oryza brachyantha*	0.26	1.39	1.64	0.30	0.27	-0.25	-0.11	1.57
04917	WRKY transcription factor	*(Populus tomentosa x P*. *bolleana) x P*. *tomentosa*	0.94	1.39	-0.03	0.18	0.24	0.02	-0.24	-0.35
00414	NAC protein 1	*Elaeis guineensis*	2.87	1.37	0.57	0.65	0.42	0.45	-0.71	-0.51
00062	polyphenol oxidase	*Musa acuminata* AAA Group	0.61	1.33	0.15	0.03	-0.09	0.57	0.74	1.37
01550	tau glutathione S-transferase	*Allium cepa*	3.13	1.32	0.34	0.83	0.57	0.64	0.33	1.07
HA66E9C01BFEF6	glutathione S-transferase	*Camellia japonica*	1.66	1.30	1.24	2.30	0.35	0.60	0.24	-0.50
06497	PREDICTED: expansin-like B1-like	*Solanum tuberosum*	1.92	1.28	0.15	0.53	0.48	0.01	-1.18	-0.20
00648	l-asparaginase, putative	*Ricinus communis*	1.43	1.24	0.74	0.78	-0.12	-0.72	-0.71	-0.38
03083	arabinogalactan-protein	*Pyrus communis*	0.28	1.23	-0.10	0.51	-0.43	-1.30	1.03	0.94
00168	unnamed protein product	*V*. *vinifera*	1.51	1.21	0.30	0.05	0.31	-0.36	0.05	0.21
02366	oxidoreductase	*Arabidopsis lyrata* subsp. *lyrata*	4.33	1.19	0.49	0.73	0.00	0.76	0.03	0.13
06952	PREDICTED: expansin-like B1	*V*. *vinifera*	1.86	1.16	0.22	0.45	0.48	0.04	-0.88	-0.16
01827	glutathione transferase5	*Zea mays*	2.89	1.16	0.39	0.56	0.15	0.14	0.47	0.72
HA66E9C01AN9EE	peroxidase precursor, partial	*Glycine max*	-0.05	1.13	-0.01	0.72	-0.96	-0.70	2.06	1.70
02861	Os01g0847700	*Oryza sativa* Japonica Group	3.67	1.12	0.29	0.94	0.09	0.74	-0.42	0.00
03030	asparagine synthetase	*Asparagus officinalis*	0.94	1.10	-0.18	-0.83	-0.96	-0.09	2.32	0.42
HA66E9C01BDZ6K	universal stress protein A-like protein	*V*. *vinifera*	-0.25	1.10	-0.05	0.16	-0.09	0.26	0.05	-0.11
02288	MLP-like protein 423-like	*S*. *tuberosum*	0.16	1.09	-0.40	1.00	-1.12	-1.16	1.69	1.43

The 25 most significant transcripts (Log_2_FC ≥1.0 or ≤-1.0) induced by *Pc* were matched against the SA and MeJA treatments. Genes induced by either phytohormone were contrasted against those induced by *Pc* to identify which infection-induced genes were SA or MeJA linked. Significant fold changes (≥1.0) are indicated in green while transcripts repressed (≤-1.0) are indicated in red. Salicylic acid (SA), methyl jasmonate (MeJA), *Phytophthora cinnamomi* (*Pc)*.

**Table 3:** Top 25 avocado transcripts differentially regulated by *Phytophthora cinnamomi* (*Pc*) at 18 hours post infection.

Sequence ID	Putative identification	Organism	6 hrs			18 hrs			24 hrs	
			SA	*Pc*	MeJA	SA	*Pc*	MeJA	*Pc*	MeJA
06520	Cysteine-rich repeat secretory protein	*Medicago truncatula*	0.01	0.53	0.75	1.62	2.09	0.50	-2.62	-0.49
01242	PREDICTED: universal stress protein A-like protein	*Vitis vinifera*	-1.36	-1.04	-1.67	-0.07	1.96	1.71	-0.01	-0.44
05905	PREDICTED: early nodulin-75-like	*V*. *vinifera*	-0.88	0.28	-1.26	0.16	1.90	-0.20	-1.27	-0.88
07563	Uncharacterized protein LOC100801029	*Glycine max*	-1.51	-1.18	-4.22	-2.16	1.66	0.95	-0.21	-0.75
02037	asparagine synthetase, putative	*Ricinus communis*	0.53	-0.16	0.29	0.82	1.61	1.08	-1.22	0.19
01014	class I chitinase	*Picea engelmannii x Picea glauca*	-1.47	-0.89	-0.61	0.94	1.60	1.33	-2.27	-1.08
02831	type 2 ribosome-inactivating protein cinnamomin II precursor	*Cinnamomum camphora*	0.46	0.04	-1.16	0.55	1.34	1.58	-1.53	-0.39
02817	PREDICTED: basic 7S globulin	*V*. *vinifera*	-1.22	-0.90	-1.18	0.95	1.34	0.95	-2.07	-0.97
03182	receptor-like protein kinase	*Populus trichocarpa*	0.14	0.24	0.57	1.14	1.27	0.24	-2.63	-0.64
01261	class I chitinase	*P*. *engelmannii x P*. *glauca*	0.14	-0.06	0.40	1.22	1.21	0.99	-1.55	-1.23
00535	endochitinase	*Persea americana*	0.06	0.28	-0.39	1.67	1.18	1.14	-2.31	-0.92
05213	protease inhibitor-like	*G*. *max*	-1.38	-0.94	-1.53	0.51	1.16	1.09	-0.90	-0.48
01782	ERF-like protein	*Cucumis melo*	0.37	0.36	-0.36	0.37	1.15	0.42	-1.14	-0.62
01428	uncharacterized protein LOC100262584	*V*. *vinifera*	-0.22	-0.05	-0.45	0.52	1.13	0.20	-1.17	-0.50
05493	conserved hypothetical protein	*R*. *communis*	0.24	-0.04	0.22	0.26	1.10	-0.05	0.08	-0.04
00542	AF239617_1beta-1,3-glucanase	*V*. *vinifera*	-1.75	-0.84	-0.74	1.51	1.09	1.37	-2.02	-0.51
00740	*leucoanthocyanidin dioxygenase*	*P*. *trichocarpa*	-0.01	0.52	1.57	1.00	1.05	0.58	-1.85	0.27
GI32N0T02G4K2R	disease resistance RPP13-like protein 4	*V*. *vinifera*	0.02	-0.03	0.09	1.17	1.05	0.07	0.08	-0.05
02171	MFP1 attachment factor 1-like	*Solanum tuberosum*	-0.36	0.13	-0.12	0.30	1.04	-0.16	0.02	-0.08
01794	uncharacterized protein LOC100248895	*V*. *vinifera*	-0.01	-0.77	0.38	0.70	1.03	1.18	-0.26	-0.16
06594	Unknown	Unknown	0.33	0.72	0.68	0.34	1.28	0.29	1.20	0.44
GI32N0T02GDB8Q	peroxidase 51-like precursor	*S*. *tuberosum*	-0.07	0.29	-0.09	1.44	1.03	0.35	-1.21	-0.39
00475	nodulin MtN3 family protein	*P*. *trichocarpa*	-0.66	-0.21	-0.78	0.36	1.02	0.26	-0.25	0.03
01593	pathogenesis-related protein 1	*Musa acuminata*	0.37	0.43	0.10	1.71	1.01	1.37	-2.18	-1.23
02540	PREDICTED: miraculin	*V*. *vinifera*	-0.52	-0.31	-0.05	0.59	1.01	0.25	-1.38	-0.24

The 25 most significant transcripts (Log_2_FC ≥1.0 or ≤-1.0) induced by *Pc* were matched against the SA and MeJA treatments. Genes induced by either phytohormone were contrasted against those induced by *Pc* in order to identify which infection-induced genes were SA or MeJA linked. Significant fold changes (≥1.0) are indicated in green while transcripts repressed (≤-1.0) are indicated in red. Salicylic acid (SA), methyl jasmonate (MeJA), *Phytophthora cinnamomi* (*Pc*).

**Table 4:** Top 25 avocado transcripts differentially regulated by *Phytophthora cinnamomi* (*Pc*) at 24 hours post infection.

Sequence ID	Putative identification	Organism	6 hrs			18 hrs			24 hrs	
			SA	*Pc*	MeJA	SA	*Pc*	MeJA	*Pc*	MeJA
06179	dihydroflavonol 4-reductase	*Epimedium sagittatum*	-0.72	0.02	-0.01	0.45	-0.77	-1.57	2.45	1.69
04939	PREDICTED: metallothionein-like protein-like isoform 1	*Vitis vinifera*	0.30	1.91	0.34	1.53	-0.02	-2.06	2.44	2.52
04303	AF279655_1metallothionein-like protein	*Typha latifolia*	0.36	1.91	0.38	0.90	-1.06	-2.39	2.33	2.40
03030	asparagine synthetase	*Asparagus officinalis*	0.94	1.10	-0.18	-0.83	-0.96	-0.09	2.32	0.42
HA66E9C01AN9EE	peroxidase precursor, partial	*Glycine max*	-0.05	1.13	-0.01	0.72	-0.96	-0.70	2.06	1.70
04599	sesquiterpene synthase	*Azadirachta indica*	-0.51	0.35	-0.72	0.54	-1.24	-1.74	2.02	1.44
01429	dihydroflavinol reductase	*Dendrobium moniliforme*	-0.77	0.42	-0.33	0.20	-1.08	-2.03	2.00	1.39
00387	delta-12 fatty acid desaturase	*Persea americana*	-0.43	0.10	-0.67	0.15	-1.02	-1.25	1.90	1.26
02288	PREDICTED: MLP-like protein 423-like	*Solanum tuberosum*	0.16	1.09	-0.40	1.00	-1.12	-1.16	1.69	1.43
05744	chalcone synthase	*P*. *americana*	-0.77	0.53	-0.23	0.10	-1.11	-2.13	1.63	1.18
07016	chalcone synthase	*Acer palmatum*	-0.73	0.60	-0.04	0.27	-1.08	-2.00	1.61	1.25
02593	PREDICTED: SPX domain-containing protein 2-like	*Solanum lycopersicum*	-2.29	-2.11	-0.01	-1.58	-2.38	-1.34	1.56	0.63
GI32N0T02IP903	peroxidase 16 precursor family protein	*Populus trichocarpa*	-0.43	0.42	-0.81	0.57	-0.69	-0.30	1.43	1.17
07665	chalcone synthase	*V*. *vinifera*	-0.75	0.54	-0.28	-0.03	-1.11	-2.05	1.42	1.08
01438	chalcone isomerase	*Gossypium hirsutum*	-0.47	0.35	-0.07	0.34	-0.80	-1.30	1.40	1.13
GI32N0T02GTAO6	G1P adenylyltransferase large subunit 2	*Cicer arietinum*	-0.10	0.31	-0.45	-0.69	-0.45	-0.83	1.32	1.02
03823	lipid binding protein, putative	*Ricinus communis*	-1.03	0.13	-0.75	0.03	-0.05	-0.69	1.31	0.75
01186	class IV chitinase	*Pseudotsuga menziesii*	-1.18	0.09	-0.61	0.35	-0.66	-0.81	1.23	1.45
HA66E9C01BJOK1	PREDICTED: phospholipase C 3-like isoform 1	*V*. *vinifera*	-1.75	-1.48	-0.45	-0.85	-1.71	-0.45	1.15	0.53
01026	polyphenoloxidase	*Camellia nitidissima*	0.66	1.88	0.61	0.08	-0.30	0.96	1.15	1.93
01719	Basic blue protein	*Medicago truncatula*	1.12	1.53	-0.50	-0.38	0.97	0.28	1.13	0.96
06807	cotton annexin 6	*Gossypium hirsutum*	0.23	0.53	0.16	0.36	-0.23	-0.39	1.11	1.35
HA66E9C01BHOQ7	carotenoid cleavage dioxygenase 2	*Crocus sativus*	-1.08	-0.37	-0.31	0.35	-0.06	-0.62	1.09	0.41
05878	hypothetical protein ARALYDRAFT_493374	*Arabidopsis lyrata* subsp. *lyrata*	-0.90	-0.40	-1.06	-0.24	-0.23	0.06	1.08	-0.09
03333	arabinogalactan protein	*Daucus carota*	-0.07	0.87	-0.24	0.72	-0.25	-0.46	1.07	1.32

The 25 most significant transcripts (Log_2_FC ≥1.0 or ≤-1.0) induced by *Pc* were matched against the SA and MeJA treatments. Genes induced by either phytohormone were contrasted against those induced by *Pc* in order to identify which infection-induced genes were SA or MeJA linked. Significant fold changes (≥1.0) are indicated in green while transcripts repressed (≤-1.0) are indicated in red. Salicylic acid (SA), methyl jasmonate (MeJA), *Phytophthora cinnamomi* (*Pc*).

#### Transcription factors and auxiliary pathways

We observed a significant induction of defense-related transcription factors upstream of phytohormone signaling pathways such as the *WRKY transcription factor* (04917), *NAC protein 1 transcription factor* (00414) and *annexin D5* (02842) which were differentially regulated in response to SA and *Pc* at 6 hpt (**Table 2**). *NAC protein 1* (00414) regulates signaling pathways and pathogen interactions [40] and represses or induces *PR* gene expression by interacting with phytohormone signaling pathways [41]. Functional annotation of the *NAC protein* is required to elucidate its role in the avocado-*Pc* interaction. *WRKY* TF (04917) was induced by *Pc* infection. These TFs are plant specific, contain a conserved peptide sequence (WRKYGQK) and a zinc finger motif and may be involved in SA signaling [42–45]. Multiple studies have shown that TFs stimulate plant defense signaling pathways in different directions [46].

Plant annexins are Ca^2+^ and phospholipid-binding proteins induced in response to pathogens, abiotic stress, SA- and MeJA- treatment(s) [47, 48]. *Annexin D5* was upregulated at 6 hpi demonstrating the involvement of Ca^2+^ signal transduction *en route* to defense gene activation. The Ca^2+^ influx of ions is induced by MAMPs, effectors, and hormones, perceived by Ca^2+^-dependent protein kinases (CDPKs) and results in signal transduction and activation of other signaling pathways and induction of *PR* gene(s) [33, 49]. *Annexin D5* induction continued at 18 hrs post SA treatment but not at 18 hrs post *Pc* infection. Ca^2+^- signaling transcripts, *cotton annexin 6* (06807) and *delta-12 fatty acid desaturase*, (00387) were linked to JA signaling and up-regulated at 24 hpi by the pathogen (**Table 4**), a time-point where infection is already well established according to previous studies [8, 20]. These genes are not important in the initial biotrophic responsive stages of infection and could be linked to the activation of JA signaling, which we believe is induced when the pathogen switches from the biotrophic life stage to necrotrophy.

#### Secondary metabolite production

Secondary metabolites are important in various biotic and abiotic stress responses and perform a broad array of protective functions, including antimicrobial, structure stabilizing and signaling and detoxification of ROS [50]. Several transcripts coding for secondary metabolites were identified across all time points with the majority occurring at 18 hpi. Detoxification enzymes such as glutathione transferases (GSTs) (01550, 01827, HA66E9C01BFEF6), oxidoreductases (02366), peroxidase (GI32N0T02GDB8Q, GI32N0T02IP903 and HA66E9C01AN9EE) and metallothioneins (04939, 04303) were elevated by both SA application and *Pc* infection (**Tables 2, 3 and 4**).

In our expression data, eight GSTs showed induction in response to SA (01422, 02020, 01550, 01568, 01577, HA66E9C01A94Q0, HA66E9C01ANAPC, HA66E9C01BFEF6) of which three were also induced by *Pc* at 6 and 18 hpi (01550, 01827, HA66E9C01BFEF6) (**Table 2**), while one was induced only by *Pc* at 24 hpi (14031). GSTs play a role in detoxification and plant stress [51] by facilitating conjugation of the toxicant with glutathione and neutralizing toxic components released by both plants and pathogens. In tomato, *GST* transcripts were enriched in the roots of plants treated with SA [52]. These enzymes have been implicated in defense against *Pc* in the non-host interaction with maize [53] and were also found to be induced at 6 hpi in this study. A clear difference between the maize-*Pc* and the avocado-*Pc* interaction is the expression of *GST* transcripts at 24 hpi. In the resistant maize interaction, *GST* expression already ceased by 24 hpi whereas in the resistant avocado interaction *GST* expression remains upregulated (**Table 2**).

The *oxidoreductase* (02366) upregulated at 6 hrs in response to *Pc* (**Table 2**) contains the aldo-keto reductase (AKR) domain and participates in detoxification, functions against pathogen attack and is involved in plant secondary metabolic pathways including flavonoid biosynthesis [54]. The up-regulation of *oxidoreductase* at 6 hpi in avocado roots indicates the induction of the SA signaling pathway and possibly that of flavonoid biosynthesis in response to infection [49].

The role of flavonoids in avocado defense against *Pc* is supported by the identification and regulation of *chalcone isomerase* (01438) and *leucoanthocyanidin dioxygenase* (00740). Chalcone isomerase is an important enzymes of flavonoid biosynthesis and is involved in the SA defense pathway [55] although JA- and MeJA- induction has also been reported [56, 57]. Our study shows that *chalcone isomerase* (01438) was MeJA responsive and was also induced by *Pc* at 24 hpi (**Table 4**). *Leucoanthocyanidin dioxygenase* (00740) expression was elevated by both SA and *Pc* at 18 hrs (**Table 3**). It catalyzes the conversion of leucoanthocyanidin to anthocyanidins in the anthocyanin pathway and is linked with environmental stress responses [58]. Genes involved in the anthocyanin pathway are involved in antioxidant activity and are induced in response to environmental stress [58] and pathogens [59].

From 18 to 24 hpi, additional *peroxidase* transcripts such as the SA-inducible *peroxidase 51-like precursor* (GI32N0T02GDB8Q) and JA-inducible transcripts of *peroxidase precursors* (GI32N0T02IP903 and HA66E9C01AN9EE) were upregulated (**Table 4**). In addition to the defense role of ROS, they also act as signaling molecules [60]. When functioning as signaling molecules, ROS is elevated along with an increase in intracellular Ca^2+^ [61]. Combined with the TF and Ca^2+^ signaling activity, the ROS activity observed at 6 hpi may be linked to ROS signaling. At 18 and 24 hpi however, increased ROS formation may function in the generation of defense-related oxygen species.

Metallothioneins are low molecular weight antioxidants [62]. The expression of SA-inducible *metallothionein-like protein-like isoform 1* (04939) was high in *Pc*-infected samples at 6 and 24 hpi (**Table 2**) and another MeJA-inducible *metallothionein-like protein* (04303) was elevated at 24 hpi (**Table 4**). Although their role is not fully understood, they are important in detoxification systems in plants and are clearly expressed during pathogen infection [8, 62, 63]. Previously we identified a putative *metallothionein-like* transcript induced at 12 hrs post *Pc* infection [8]. Sequence-comparisons showed no similarities and thus two additional putative metallothioneins are now known to be involved in the defense response to *Pc*.

#### Cell wall strengthening

Cell wall reinforcement is a basic defense response and is frequently observed in plants [64, 65]. Avocado defense mechanisms have been shown to include structural responses such as cell wall strengthening by the deposition of callose and lignin [66]. While the callose synthase gene transcripts were not among those differentially expressed, genes involved in lignin formation were induced from 6 hpi. The *basic blue protein* (01719) is a copper binding protein implicated in redox reactions during primary defense and lignin formation [67], and was significantly induced at 6 hpt in response to SA as well as at 6 and 24 hpi with *Pc*, respectively (**Table 2**). Its involvement in the pathogen response is possibly linked to cell wall strengthening [34, 68]. The involvement of cell wall modification in response to *Pc* infection is further enforced by the induction of two SA-inducible *expansin-like B1* genes (06497 and 06952) at 6 hpi (**Table 2**). Expansins loosen cell wall proteins during growth and have previously been shown to be an important response to pathogenesis of necrotrophic pathogens [69]. It has been hypothesized that they function by disrupting the bonding of the glycans to the microfibril surface [70].

*Dihydroflavonol 4-reductase* (06179) and *dihydroflavinol reductase* (01429) were elevated at 24 hrs in response to MeJA and *Pc* (**Table 4**). These genes represent enzymes that participate in lignin biosynthesis in woody plants [71, 72]. Their elevation in avocado at 24 hpi indicates an increased effort in an attempt to localize the pathogen by strengthening plant cell walls with lignin.

*Arabinogalactan*, although significantly induced at 24 hpi, showed an increase (0.8-fold) as early as 6 hpi. Two *arabinogalactans* (03083; **Table 2**) and (03333; **Table 4**) were upregulated in response to *Pc* and SA at 6 hrs and *Pc* and MeJA at 24 hrs, respectively. Arabinogalactans are glycosylated plant cell wall proteins implicated in root-microbe interactions [73] and provide protection against oomycetes by immobilizing zoospores, reducing cyst germination and hyphal proliferation [74]. *Pc* zoospores are present on avocado roots at 3 hrs [66], and the early response with *arabinogalactan* could be responsible for the decrease in encystment that has been reported on Dusa. The upregulation of arabinogalactan at 24 hpi could further contribute to the resistance by preventing colonization. Earlier elevation in the expression of *arabinogalactan* could be beneficial in combating *Pc*. Due to its action in the immobilization of zoospores arabinogalactan presents as an exciting target for further investigation into protection against *Pc*.

#### Defense associated genes

Several genes previously reported to play a role in defense in other plants against oomycetes were also upregulated in avocado roots in response to *Pc*. *PR1* (01593) expression was induced at similar levels by all treatments at 18 hrs (**Table 3**). *PR1* is induced by SA [75] but also by the JA pathway [76]. PR1 antifungal activity has previously been demonstrated against oomycetes [77, 78], and our results suggest that it is important in avocado defense against *Pc*. Another *PR1* homolog, *peroxidase 51-like precursor* (GI32N0T02GDB8Q) was also up-regulated at 18 hrs by SA, MeJA and *Pc* (**Table 3**). Other defense-associated transcripts such as *PR4* (01395) and *PR10* (contig01285) were present but not significantly expressed.

At 18 hpi a significant increase in expression (in both *Pc* and SA datasets) of a putative *R-gene*, *disease resistance RPP13-like protein 4* (GI32N0T02G4K2R), was observed (**Table 3**). The *disease resistance RPP13-like protein 4* belongs to the NBS-LRR protein family and contains leucine rich repeats (LRR) and nucleotide binding regions [79]. The LRR region of these NB-LRR receptors may be responsible for gene-for-gene specificity in the *R*-*Avr* interaction [68]. The *RPP13* allele from *A*. *thaliana* confers resistance to five different isolates of the biotrophic oomycete, *Hyaloperonospora parasitica* [80] and RPP13-like proteins are upregulated in many other plant-pathogen interactions [81–83]. Dusa® does not have complete resistance against *Pc* possibly due to the pathogen evolving a mechanism to repress RPP13 activity via effectors [42]. Lastly, based on the plasticity of the defense response and the role of R proteins functioning as MAMPs as suggested by the invasion model [84], it could be hypothesized that *RPP13* is a component in the defense machinery of avocado leading to successful defense. Discovering the Avr counterpart of RPP13 in *Pc* will be useful in resolving the function.

#### Cell wall degradation of the pathogen

Plant chitinases are produced during pathogen infection and accumulate extracellularly in infected plant tissue. At 18 hrs, e*ndochitinase* (00535) was significantly induced by both phytohormone treatments and *Pc* infection, followed by a significant down-regulation (2.3 fold) at 24 hpi (**Table 3**). The potato-*P*. *infestans* interaction [85], *P*. *cinnamomi-Z*. *mays* interaction [53], and avocado-*Pc* interaction [86] have all reported the up-regulation of *endochitinase*. Since oomycetes have cellulosic cell walls and are thus immune to the action of chitinases, which randomly cleave chitin chains [87], the expression of *endochitinases* is a curious response to *Pc*. We hypothesise that the upregulation of *endochitinases* form part of a general defense response. At 18 hrs, *class I chitinase* (01014) and *β*-*glucanase* (00542, 03461), were up-regulated by SA, MeJA and *Pc* (**Table 3**). By 24 hpi the only JA-associated cell wall digestive enzyme found was a *class IV chitinase* (01186) (**Table 4**).

*β-1*,*3-glucanases* act directly on fungal pathogens by degrading β -1,3/1,6-glucans and function in combination with chitinase [88]. The *Pc* cell wall comprises of cellulose [1] which is a β-1,4-glucan [89] and therefore *β-1*,*3-glucanases* would be ineffective. However, it has been reported that *Phytophthora* cell walls are made up of β-1,3- and β-1,6-linked glucans [90] and electron microscopy of *P*. *infestans* in potato showed that a β-1,3-glucan-specific antibody labelled callose occurred in the *P*. *infestans* cell walls indicating the presence of β-1,3-glucan bonds. Thus, *β-1*,*3-glucanases* may be an important factor limiting pathogen spread by negatively effecting hyphal cell walls.

#### Participation of other proteins in disease resistance

Transcripts grouped into this category are not generally associated with disease resistance, but some have been reported to be involved in the response to pathogens [91–93]. At 6 hpt, SA-mediated up-regulation of *l-asparaginase* (00648), *glutaredoxin* (03730) and *thioredoxin* (02211) transcripts were observed along with the JA-associated up-regulation of *polyphenol oxidase* (05008, 01026) (**Table 2**). Polyphenol oxidases (PPOs) are oxidative enzymes involved in browning of fruit through the production of reactive quinone products whose activity requires molecular oxygen [94]. Correlations between *polyphenoloxidase* expression and defense reactions are well established, however the mode of action remains unclear [93]. *Polyphenoloxidase* induction at 6 hpi implies its importance in the early defense against *Pc*. The *type 2 ribosome-inactivating protein cinnamomin II precursor* (02831) was induced at 18 hrs by MeJA and *Pc* (**Table 2**). These proteins play a role in plant defense with functions as diverse as chitinase, superoxide dismutase and lipase activities [95]. Due to the activation of other chitinase and ROS transcripts we assume that the function of this transcript could possibly fall into one of these groups.

#### Other signaling pathways implicated in the avocado- *P*. *cinnamomi* interaction

The auxin and ABA signaling pathways were implicated in the avocado- *Pc* interaction by GO classification. *Alcohol dehydrogenase* (GI32N0T02GV1IP) was both MeJA- and pathogen responsive at 18 hrs (1.1 fold- change induction). The involvement of ABA in the avocado defense response against *Pc* was identified in the GO analysis and could very well be linked to the induction of *alcohol dehydrogenase* [96]. *Aminocyclopropane-1-carboxylate (ACC) oxidase* (00641) and the *ethylene response factor* (*ERF) -like protein* (01782) were significantly repressed at 24 hpi (**Table 3**). Both these genes are associated with ET signaling. ACC oxidase catalyzes the conversion of 1-aminocyclopropane-1-carboxylic acid to ET, which is the last step in ET biosynthesis, while ERF transcription factors function in signal crosstalk between JA and ET pathways [97]. Eighteen putatively described ACC oxidase transcripts were identified but showed no differential induction or repression across treatments or time-points except at 24 hpi. The repression of the ET pathway in avocado is interesting as *ACC oxidase* was induced 6 hpi in the resistant interaction between *Z*. *mays* and *Pc* [53], suggesting that ET signaling was important in the defense response of maize. The expression of *12*-*oxo*-*phtyodienoate reductase* (00651), an important enzyme in the synthesis and signaling of JA [98, 99], is elevated in response to both SA and *Pc* at 6 hrs (2.4 and 1.1 fold-change induction, respectively). The up-regulation of *12*-*oxo*-*phtyodienoate reductase* corroborates the fact that SA and JA pathways often overlap and function in unison [100, 101]. The induction of the SA and JA signaling pathways in combination with other signaling molecules demonstrates the complex crosstalk of signaling [102]. In the *Plasmopara viticola* -grapevine interaction both SA and JA/ET pathways were synergistically involved in the disease resistance. In addition to SA and JA pathways, there was evidence of ET and ABA pathways being involved in the resistance response [39]. Studies in rice also point to the involvement of more than just the SA pathway in defense responses [38].

### Model of SA and JA temporal regulation during the first 24 hrs following *P*. *cinnamomi* inoculation of avocado

**Fig 5:**
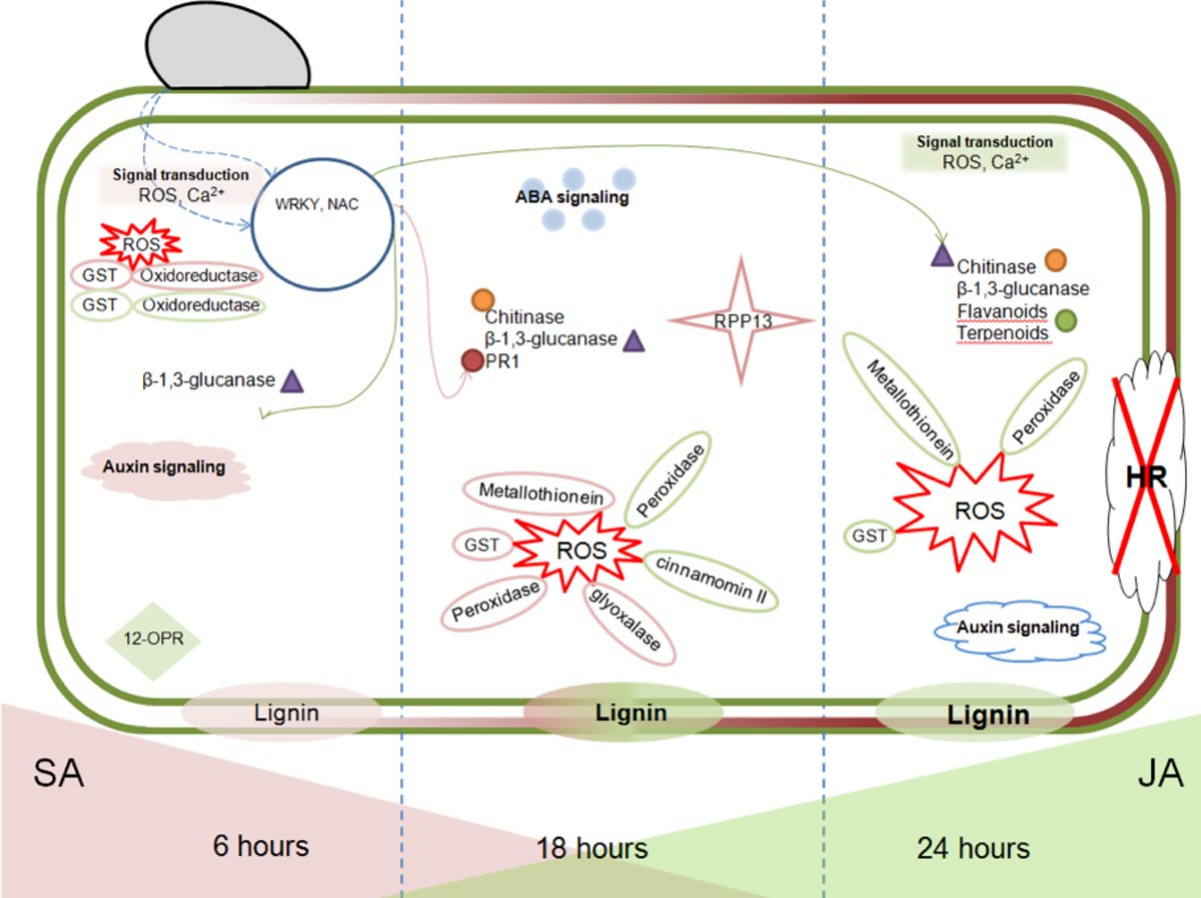
Model of SA and JA pathway temporal regulation induced by *Phytophthora cinnamomi* in tolerant avocado roots. At 6 hrs an induction of signaling pathways and transcription factors occurred, which was linked to SA signaling. Subsequently at 18 hrs, both SA and JA-linked induction of ROS, cell wall strengthening and PR transcripts were observed. At 24 hrs transcripts related to lignin formation, flavonoid and volatile compound generation, as well as JA-linked ROS were up regulated. Text shaded in pink are SA-linked, text shaded in green are JA-linked. The pathogen is indicated in grey, nucleus in blue and lignin deposition in maroon.

We propose a novel model to illustrate the initial 24 hrs following *Pc* infection of avocado roots, accounting for observations made in this study, such as cell wall strengthening, ROS production and signaling (**Fig 5**). Inoculation of avocado roots by *Pc* initiates a biphasic defense response that firstly employs SA-dependent gene expression (6 hpi), followed by JA-mediated gene expression at 18 and 24 hpi. This regulation of phytohormone pathway activity has been observed in another hemibiotrophic interaction in wheat [16]. When *F*. *graminearum* infected wheat, SA and Ca^2+^ signaling pathways were activated during the first 6 hpi followed by JA signaling around 12 hpi.

At 6 hrs following *Pc* infection, SA-mediated defense responses were active against the pathogen, lignin biosynthesis was transcriptionally elevated and there was evidence of auxin, ROS and Ca^2+^ signaling. Previously, cell wall modifications have been shown to occur in response to ROS generation [37]. Therefore, the early observation of ROS transcripts in avocado, indicates that cell wall modifications may be an important primary response to the pathogen. Our results indicate that the initial stages of *Pc* infection of avocado (6 hrs) were congruent with the study of the soybean- *P*. *sojae* interaction, which reported a predominantly SA dependent defense strategy [18]. The authors suggest that soybean encounters difficulty in switching from SA-mediated to JA-mediated defense responses, and this allows continued infection to occur without cessation [18]. We observed JA associated expression occurring at 18 hpi and becoming predominant at 24 hpi.

By 18 hpi we observed ROS, detoxification processes, cell wall reinforcement and offensive tactics employed by avocado with genes such as *β-1*,*3-glucanase*, *endochitinase* and *PR1* induced by both phytohormone treatments, indicative of an overlap between SA and JA pathways at 18 hrs. β-1,3-glucanases may be an important factor in partial digestion of the pathogen cell wall leading to activation of other defense proteins in a microbe associated molecular pattern (MAMP) triggered immunity (MTI) mediated fashion. β-1,3-glucanase activity has previously been demonstrated to be higher in tolerant/resistant avocado rootstocks when compared to less tolerant rootstocks [66]. Induction of these genes early during infection may lead to a more rapid and effective defense against *Pc*.

At 24 hpi phenolic formation in addition to the cell wall strengthening responses were induced. The majority of genes induced at this time point were linked to JA responses although it is important to note that we did not investigate SA induced material at this time-point. A study which investigated the response of wheat to infection by the hemibiotrophic pathogen *F*. *graminearum*, showed a biphasic signalling response with SA pathways being activated as early as 3 hai and then tapering down after 6 hai [16]. Based on the results from the 6 and 18 hour time-points analyzed, a reduction in SA linked defenses was anticipated and we hypothesized that by 24 hpi the involvement of the SA pathway would be minimal. The activation of the JA pathway over that of SA induced defense has been observed in non-host interactions such as the *Arabidopsis- Pc* interaction [10] and more recently in the non-host interaction of *Z*. *mays* and *Pc*. In *Z*. *mays*, resistance was associated with the early induction of the JA pathway at 6 hpi [53]. It appears that the non-host strategy involves inducing the JA defense pathway prior to that of SA. When considering this along with the findings from the wheat-*F*. *graminearum* phytohormone study [16], it appears that the timing of JA pathway induction could play a pivotal role in resistance to *Pc*.

## Conclusions

The elucidation of a host-pathogen interaction at the molecular level is necessary to understand the activity of interconnected signaling networks [103]. The transcriptome wide analysis of an incompatible avocado-*Pc* interaction has uncovered the successful employment of SA- and JA associated genes to inhibit the hemibiotrophic oomycete. We provide evidence that SA mediated defense occurs at 6 hpi and tapers off at 18 hpi followed by the enhancement of JA mediated defense from 18 hpi to 24 hpi. In addition to this auxin related genes were increased by the pathogen at 6 hpi and 24 hpi while ABA related genes were found at 18 hpi. The expression of microbial cell wall digesting enzymes and defense genes were heightened at 18 hpi while plant cell wall strengthening was predominantly elevated at 24 hpi. Other interesting findings included the elevated expression of *arabinogalactan* homologs at 6 and 24 hpi, and the *RPP13 R gene* at 18 hpi. An understanding of the effects of phytohormone activity and their associated defense responses will allow their use in manipulating defense responses *in planta* and assist in combating PRR. The identification of essential defense-related genes is important for both traditional breeding and for biotechnological development of resistant avocado rootstocks which can potentially minimize the use of chemicals for *Pc* control in the future.

## Supporting information

S1 Fig RT-qPCR validations of microarray data across 6, 18 and 24 hours of *P*. *cinnamomi* infected and SA and MeJA induced samples. Fold change expression is shown for RT-qPCR data (blue diamonds) vs. fold change data of microarray data (red squares) for *PR4* (A1-A3), *JAZ3* (B1-B3), *PR1* (C1-C3), *PAL* (D1-D3) and *α1 tubulin* (E1-E3). *P*. *cinnamomi* infected samples (A1, B1, C1, D1 and E1), SA induced samples (A2, B2, C2, D2 and E2) and MeJA induced samples (A3, B3, C3, D3 and E3). Error bars indicate the SEM for three biological replicates. The Y-axis represents relative fold change and the X-axis represents the time points after treatment.
(TIFF)

S1 Table Primer sequences for RT-qPCR validation of avocado microarray data. (PDF)

S2 Table Transcripts commonly and uniquely expressed among the *P*. *cinnamomi* infected and SA and MeJA treated samples 6, 18 and 24 hours. (XLSX)
