# Supplementary figures and images for: Transcriptome analysis of an incompatible Persea americana-Phytophthora cinnamomi interaction reveals the involvement of SA- and JA-pathways in a successful defense response
Source: PLoS One. 2018 Oct 17;13(10):e0205705. doi: 10.1371/journal.pone.0205705 (PMC6192619; doi:10.1371/journal.pone.0205705)

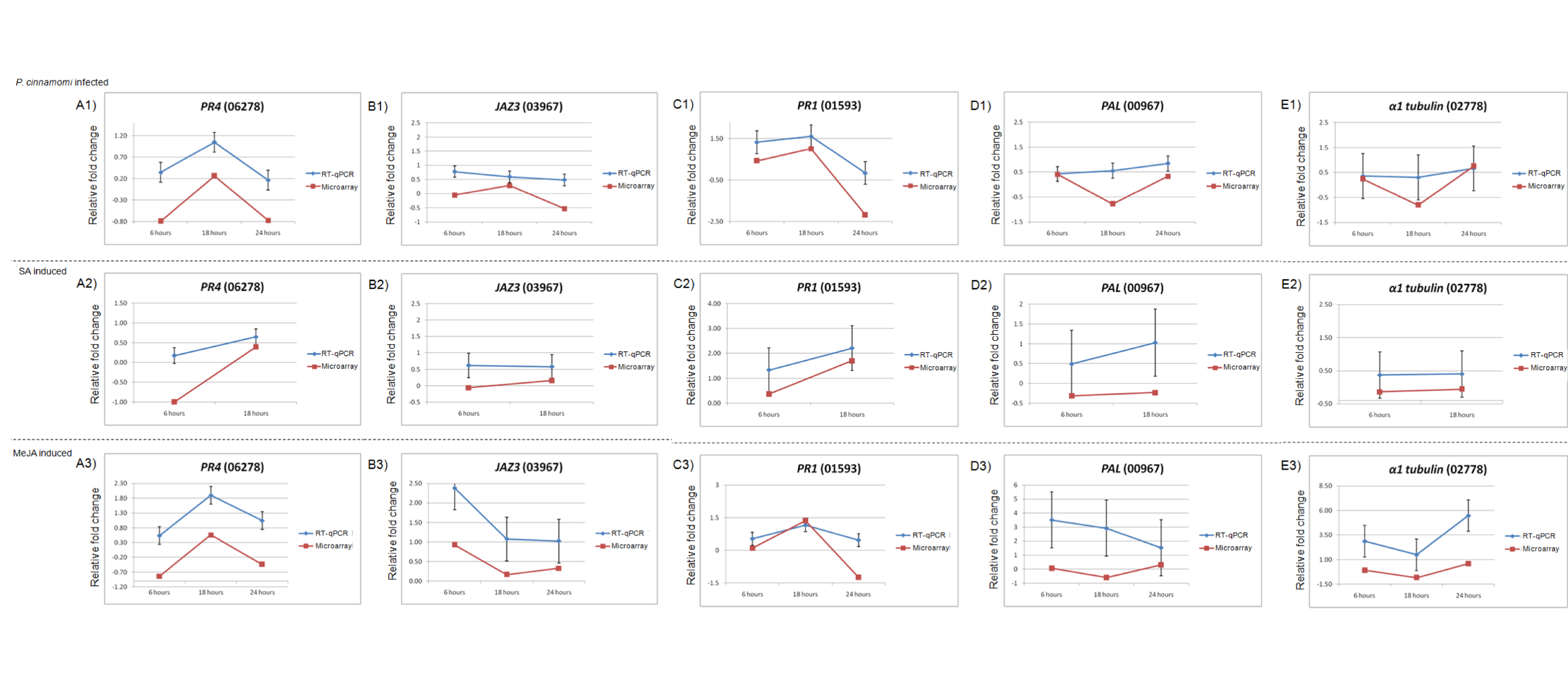

Supplement: S1 Fig — Fold change expression is shown for RT-qPCR data (blue diamonds) vs. fold change data of microarray data (red squares) for PR4 (A1-A3), JAZ3 (B1-B3), PR1 (C1-C3), PAL (D1-D3) and α1 tubulin (E1-E3). P. cinnamomi infected samples (A1, B1, C1, D1 and E1), SA induced samples (A2, B2, C2, D2 and E2) and MeJA induced samples (A3, B3, C3, D3 and E3). Error bars indicate the SEM for three biological replicates. The Y-axis represents relative fold change and the X-axis represents the time points after treatment. (TIFF) [file pone.0205705.s001.tiff]
