# Supplementary material for: Transcriptome analysis of an incompatible Persea americana-Phytophthora cinnamomi interaction reveals the involvement of SA- and JA-pathways in a successful defense response
Source: PLoS One. 2018 Oct 17;13(10):e0205705. doi: 10.1371/journal.pone.0205705 (PMC6192619; doi:10.1371/journal.pone.0205705)
Supplement: S1 Table — (PDF) [file pone.0205705.s002.pdf]

**S1 Table. Primer sequences for RT-qPCR validation of avocado microarray data.**

| Gene | Seq ID | Forward primer (5'-3') | Reverse primer (5'-3') | Product size (bp) | Annealing temperature | Efficiency % |
|---|---|---|---|---|---|---|
| ***LOX*** | 00275 | TGTCGGACCTGATCCAAACTTGC | CCCAAAGTTGACTGCATGG | 74 | 62.5 °C | 99 |
| ***PR4*** | 06278 | GACAGGAGCTCAGACAACAGTGAG | AGCCACCCTCATCCAAATCCAG | 74 | 58 °C | 92 |
| ***PR1*** | 01593 | GCGGCTGGAAAGGTTTGT | GGGGCTGTAGTTGCAAGT | 102 | 58 °C | 97.6 |
| ***JAZ3*** | 03967 | GCGATGGTGAAAGAGGAGAC | TGAAATGAGGAAGAGTTGAGACC | 108 | 59.5 °C | 95 |
| ***α1 tubulin*** | 02778 | GGATGTAGTCTTCTCTGTTCCC | CAGTATGCCATTACCATGAATGC | 122 | 58 °C | 91.8 |

Primers were designed for *lipoxygenase 1* (*LOX*), *pathogenesis-related group-4* (*PR-4*), *pathogenesis-related group-1* (*PR-1*), *jasmonate ZIM-domain* (*JAZ3*) genes. *α1 tubulin* was used as a reference gene. All genes had amplification efficiencies above 90% and amplified between 58 and 62.5°C.
